# Cockleburr in the Air Passage of a Child Successfully Removed by an Intra-Laryngeal Operation

**Published:** 1893-01

**Authors:** W. L. Bullard

**Affiliations:** 1408 3rd Ave.; Columbus, Ga.


### COCKLEBURR IN THE AIR-PASSAGE OF A CHILD SUCCESSFULLY REMOVED BY AN INTRA-LARYNGEAL OPERATION.

BY DR. W. L. BULLARD, COLUMBUS, GA.

Some days ago, Mr. W. J. B. F., of Cottage Milles, Ga., brought to my office his little boy, age ten years, accompanied by the family physician, Dr. Sapp, saying that the little fellow had on the day before, while driving cows through a field in which there were cockleburr weeds, in some way inhaled a burr into the trachea. The little fellow's breathing was labored; the wheezing, which could be distinctly heard into the reception room, was very distressing to other patients therein assembled. As is usually the case with most patients who have a foreign body in their larynx or trachea, my little patient was very excitable and apprehensive.

Quieting him as much as possible with fair promises of not giving or causing him any pain, I had him seated for a tracheascopic examination. Patient behaved admirably, and with the laryngoscope the burr was readily located and pointed out to Dr. Sapp. It had become impacted in the glottis. Realizing the immediate danger from complete obstruction, I made everything ready for laryngotomy or tracheotomy, feeling confident, however, (should the obstruction not become complete) that I would be able to deviate from the general rule, that is to say, remove the corpus alienum by an intra-laryngeal operation instead of tracheotomy as advised by most text-books on surgery and practiced by the most eminent surgeons. I hastened to cocainize the pharynx and larynx with a six per cent. solution of mur. cocaine, and with the co-operation of the little patient, it was not long before I had the burr safely in the grasp of a Stoerk's tube forceps and readily extracted it to the admiration of Dr. Sapp and indescribable relief to the patient and the father of the child. With some advice as to treatment should the laryngeal inflammation not soon subside, the little hero was, on the first train, carried to his home instead of having to remain in the city for a week perhaps had I performed either laryngotomy or tracheotomy.

Hamilton (1) says, "In short we have very few resources in this unfortunate class of accidents except a surgical operation. If the object for which we are to search is lodged in the ventricals of the larynx, the operation of laryngotomy is to be preferred." Agnew (2) advises, in that most classical work of his on surgery, that "When it can be determined that the body is located in one of the ventricals of the larynx, laryngotomy is to be preferred." The Atlanta Society of Medicine reports (3) the following: "Dr. Willis Westmoreland reported two cases of tracheotomy for foreign bodies. One, a girl eight years old, swallowed a piece of knitting needle bent in the shape of a hair pin. Cut down and found it without much difficulty. The second swallowed a cockleburr four days previously. Performed tracheotomy. Could not find the foreign body; cut two more rings of trachea; kept wound open and the cockleburr came out four days after operation."

I mention the above only as evidence to show that the tendency is to remove foreign bodies from the air-passages by an external operation as laryngotomy and tracheotomy, ignoring intra-laryngeal efforts, which according to my mind and experience (if the patient is in no immediate danger) should always be most diligently practiced as the most preferable method, and if found impracticable, then resort to one of the external operations.

1408 3rd Ave., December 10, 1892.

---

(1) Principles and Practice of Surgery, 1882.

(2) Principles and Practice of Surgery, 1883.

(3) Atlanta Medical and Surgical Journal, April, 1892.

---

The return of the menstrual flow after the menopause usually means malignancy, and in such cases the patients should be subjected to careful examination at once.—THOMAS.

